# Unveiling the nexus! Understanding knowledge issues, animal contact patterns and interaction of health care providers in the context of monkeypox and COVID-19 during monkeypox outbreak 2022

**DOI:** 10.1080/07853890.2024.2386452

**Published:** 2024-08-06

**Authors:** Samar A. Amer, Hossam Tharwat Ali, Sarya Swed, Omar A. Albeladi, Alex Stéphane Ndjip Ndjock, Al Zahraa M. Soliman

**Affiliations:** a Department of Public Health and Community Medicine, Faculty of Medicine, Zagazig University, Zagazig, Egypt; b Royal Colleague of General Practitioners [INT], London, UK; c Department of Mental Health Primary Care, Nova University, Lisbon, Portugal; d Qena Faculty of Medicine, South Valley University, Qena, Egypt; e Faculty of Medicine, Aleppo University, Aleppo, Syria; f Public Health Departments, King Salman Bin Abdulaziz Medical City, El Madinah, Saudi Arabia; g Department of Public Health, Edea Health District, Edea, Cameroon; h Association pour le développement de l'épidémiologie de terrain, Château de Vaccassy, Saint-Maurice Cedex, France

**Keywords:** Health care providers, information-seeking behaviour, health belief models, monkeypox, COVID-19

## Abstract

**Background:**

A monkeypox (MPOX) outbreak occurred in May 2022. On June 3, 2022, the WHO Blueprint organized a consultation on MPOX research knowledge gaps and priority research questions because the engagement of health care providers (HCPs) in providing accurate information and the public's motivation to adapt protective behaviour were crucial. Thus, we conducted this study to explore the knowledge issues, animal patterns, and interactions of HCPs in the context of MPOX and COVID-19 during the MPOX outbreak.

**Methods:**

We conducted a cross-sectional web-based survey among 816 HCPs working in governmental health facilities from many countries, mainly Syria, Egypt, Saudi Arabia, and Cameroon, in September 2022.

**Results:**

Four hundred and sixty (56.37%) were aged between 18 and less than 35 years old. About 34.44% were physicians, while only 37.25% worked on the frontlines with patients. 37.99% and 5.88% received vaccinations against chickenpox and MPOX, respectively. In the meantime, 55.39% had taken courses or training programmes regarding COVID-19. Regarding knowledge-seeking behaviours (KSBs) about COVID-19, 38.73% were through passive attention, while only 28.8% got their information through active search. Most of the participants (56.86%) had a moderate level of knowledge regarding COVID-19. Only 8.82% had courses or training programmes regarding MPOX. Regarding KSB about MPOX, 50.86% were obtained through passive attention, while only 18.01% and 23.04% got their information through active and passive search, respectively. Most of the participants (57.60%) had a poor level of knowledge regarding MPOX. The regression analysis of the MPOX knowledge score revealed that individuals working on the frontlines with patients and those who had training programmes or courses were shown to have a higher score by 1.25 and 3.18 points, respectively.

**Conclusions:**

The studied HCPs had poorer knowledge about the MPOX virus than they did about the SARS-CoV-2 virus. Training programmes and education courses had an impact on their knowledge.

## 1. Introduction

In May 2022, the World Health Organisation (WHO) convened its member states for the first in-person meeting following the COVID-19 pandemic and announced that it was no longer the only global health threat; the world faces other 'formidable' infectious diseases, including monkeypox (MPOX) [1]. On July 23, 2022, the world had not yet recovered from the COVID-19 pandemic when it was declared that the multi-country outbreak of MPOX was a public health emergency of international concern. As of August 18, 2022, 94 member states had reported 39,110 confirmed cases (including 12 deaths) of MPOX [2], and 587 million confirmed cases and 6.4 million deaths of COVID-19 had been reported globally [3].

The current MPOX is a neglected infectious zoonotic disease that is endemic in West or Central Africa. Since May 6, 2022, it has reemerged in a non-endemic country in Europe or North America without a history of travel to endemic countries [4]. It transmitted either animal-to-human or human-to-human through direct or indirect contact, or through men who have sex with men (MSM) or close intimate contact, which resulted in ongoing, unprecedented community transmissions [5–7]. Lymphadenopathies and skin cluster eruptions are the hallmarks. It is typically self-limiting, but the fatality rate varies depending on the type; for example, younger people, pregnant women, immunocompromised people, or care providers may face serious complications such as pneumonia, encephalitis, or even death, with a mortality rate of approximately 3.6% [8–13].

COVID-19 does not have a specific authorized therapy, and although 60% of the global population has received COVID-19 vaccinations, around one billion individuals are still unvaccinated. With the goal of containing the spread of MPOX, health care providers (HCPs) would typically adopt a ring vaccination strategy using the recently approved MPOX vaccines, early screening, and appropriate management, e.g., the use of the authorized antiviral medications, for the treatment of MPOX [6,12].

With the COVID-19 pandemic and MPOX outbreaks, poor and delayed health-seeking behaviour (HSB) is expected and linked to knowledge-seeking behaviour (KSB) and poor health outcomes [14]. KSB is a model of information behaviour defined as a person's inaction and procrastination with the goal of finding an appropriate remedy to restore health that also identifies information-seeking behaviour [15,16], while HSB includes the practice of preventive measures, e.g., avoiding direct contact with animals and humans that could harbour the virus or any materials that have been in contact with them; isolating infected patients from others who could be at risk for infection; vaccination; practicing proper hand hygiene; and using personal protective equipment (PPE) when indicated [17].

An essential aspect of anticipating future epidemics and implementing effective policy measures to mitigate socioeconomic consequences is comprehending the emergence, behaviour and utilization of novel viral agents. We should not underestimate the public health concerns of infectious diseases, including MPOX disease [18]. In response to this threat, WHO as well as national and local health agencies have issued an abundance of recommendations, including surveillance, contact tracking and reporting, community engagement, and distributing information to make HCPs more knowledgeable for effective clinical management [3, 11].

On June 3, 2022, the WHO R&D Blueprint organized a consultation on MPOX research knowledge gaps and priority research questions. WHO urges immediate efforts to focus on providing accurate information to people who are at risk of infection, preventing further spread among vulnerable groups, and protecting HCPs [7]. Close contact between infected patients, especially in the absence of PPE, exposes HCPs to the risk of infectious diseases (COVID-19 and MPOX). Therefore, increasing knowledge and acceptance of these preventive recommendations among HCPs is crucial [13,19].

In light of the foregoing, a novel study in many countries (mainly Syria, Egypt, Saudi Arabia, and Cameroon), which is not among those afflicted and no deaths have been documented to date, was conducted to explore knowledge issues, including KSB and level of knowledge, animal contact patterns, and the interaction of HCPs, in the context of MPOX and COVID-19, during the MPOX outbreak in 2022.

## 2. Methods

### 2.1. Participants and study design

This web-based, cross-sectional study involved 816 HCPs from more than four countries, mainly Syria, Egypt, Saudi Arabia, and Cameron. The criteria for inclusion were any HCPs, either physicians, nurses, chemists, or medical students, who worked in governmental health care facilities. We also excluded HCPs on vacation or those with complex mental, long-term, or psychiatric problems.

### 2.2. The sample

The sample size was calculated using the formula: *n* = *Z*^2^ × *P* × (1 − *P*)/*d*^2^, where *n* represents the sample size, a 95% confidence level (CI), *Zα*/2 = 1.96, a 5% margin of error, *P* is the expected prevalence, and *d* is the precision or effect size [20]. Prior to calculating the sample size, a pilot study of 40 HCPs was conducted to determine the feasibility and sample size of the study. According to the findings of this pilot study, the sample size is calculated based on the assumption of a 50% level of awareness about MPOX, an unlimited population size (due to the inability to get an accurate size of HCPs in targeted countries), a 95% CI, and a 5% margin of error. The calculated sample size is 820 HCPs using an online sample size calculator.

### 2.3. Data collection

#### 2.3.1. Design and development of the questionnaires

The survey questionnaire was developed and adapted from previous studies [17, 21, 22]. Initially written in English, it was then translated into Arabic by a bilingual team consisting of two medical professionals and one qualified outside medical translator.

#### 2.3.2. Validation of questionnaires

To ensure comprehension and cultural acceptability, a pilot test was conducted with 40 volunteers from the governmental HCPs in the four countries under study. Ten volunteers (three physicians, three nurses, and four others) from each nation completed the questionnaire. Participants rated the questionnaire's format, clarity, length, and overall assessment. Based on their feedback, certain questions were modified. The same subjects were given the questionnaire again one week later to assess reliability and repeatability. Data collected during the pilot test were excluded from the final analysis. The questionnaire demonstrated a Cronbach's alpha of 0.81.

#### 2.3.3. Questionnaire

After obtaining informed consent, participants completed and returned the questionnaire. The questionnaire consisted of the following sections: (I) sociodemographic and health-related characteristics, including age, gender, place of residence, nationality, level of education, marital status, and comorbidities. (II) KSB as a fifth group of concepts, namely passive attention, passive search, active search, and on-going search [16]. (III) The exposure and knowledge-related issues among HCPs, including the source of information, awareness, exposure to a case or suspected case, vaccination status and intentions, and the level of knowledge. The level of knowledge of the MPOX virus, COVID-19, was assessed using 21 questionnaire items adapted from another study and the CDC web site [17, 21, 22]. The possible responses to each knowledge item were (yes, and no). The score for correct responses was 1, and the score for incorrect responses was zero. The sum of these scores represented the participant's total knowledge score regarding the monkeypox knowledge score (MPOX-K score) and the COVID-19 knowledge score (COVID-19-K score). HCPs participants who obtained a score of <50% were deemed to have a poor level of knowledge, whereas those with a score of 50–75% and >75% were classified as having moderate and high levels of knowledge, respectively. (IV) The HCPs beliefs about MPOX and their adherence to health precautions (preventive and promotional health measures): through exploring the perceived susceptibility and severity of MPOX disease as well as the adherence to health preventive measures, e.g., vaccination, wearing masks, keeping safe spaces or distances, regular disinfection of surfaces, hygienic hand washing, and ticking cough etiquette [23]. We added items to measure adherence to pro-health motivational activities, e.g., drinking more than two liters of water daily, supplement intake, and regular exercise (one hour and a half per week) [24]. (V) Animal contact patterns include rodents, mice, hamsters, bats, monkeys and camels; reptile pets such as turtles, alligators, crocodiles, lizards, and snakes; aquatic pets such as fish; and avian pets such as parrots, and passerines among HCPs. The results of the experience and animal pattern of exposure sections were presented as frequencies and percentages. Nevertheless, to obtain a total score of the animal pattern of exposure, answers were recoded as follows: not at all = 0, yearly or monthly = 1, weekly = 2, and daily = 3.

#### 2.3.4. Data collection technique

Two governorate health care facilities were randomly selected from each of the targeted countries, followed by the selection of one urban and one rural area from each governorate. Official websites and social media platforms were used to distribute the questionnaire to the targeted facilities (Facebook, Twitter, WhatsApp, and Telegram groups). Snowball strategies were employed in order to collect data as professionally and objectively as possible. An author from each inquired country in our study was responsible for the data collection process, and follow-up emails and reminders were employed to increase the response rate until the desired sample size was recruited.

### 2.4. Statistical analysis

The data were organized in a Microsoft Excel (Redmond, WA) sheet and then imported and analysed using R Statistical Software (v4.1.3; R Core Team 2022) (R Foundation for Statistical Computing, Vienna, Austria). Frequencies and percentages were used to describe the categorical variables for baseline demographic characteristics. The normality of the continuous variables was assessed using the Shapiro–Wilk test. The median and interquartile range (IQR) were used to describe the parametric variables. Spearman's correlation analysis was used to assess the correlation between knowledge levels of COVID-19 and MPOX and animal contact patterns. A multivariate linear regression analysis assessed the association between demographic characteristics and knowledge level. A *p* value of ≤0.05 was considered significant.

### 2.5. Ethical considerations

All HCPs were informed of the objectives of the study, the identity of the research team, their right to withdraw from the study, and the absolute confidentiality of their personal data. All of the patients who were eligible provided written informed consent. After submitting the questionnaire, the study participants were informed about clinical signs and symptoms, as well as transmissibility and preventative measures for both diseases. Patients were also assured of their data's anonymity and confidentiality.

All participants provided written informed consent after receiving clarification regarding the study's objectives, data confidentiality, voluntary participation and the right to withdraw. The questionnaire did not include sensitive questions, and the data were collected anonymously. The study was approved by the institutional review board of the Benha University Branch of Zagazig University (BU-IRP# 9921/28-4-2022).

## 3. Results

### 3.1. Demographic characteristics

Out of the total 816 HCP participants who were included in the final analysis, 460 (56.4%) were of age between 18 and Less than 35 years old (Table 1). Egypt and Saudi Arabia came in second and third, respectively, with percentages of 22.6% and 21.7%. Syria had the highest response rate at 29.7%. Around 52% of our sample were female, while 47.8% were single and 43.3% were married. Around 34.4% were physicians, while only 37.3% worked on the frontlines with patients. The majority of HCPs (79.5%) were vaccinated against COVID-19, while only 37.9% and 5.9% were vaccinated against chickenpox and MPOX, respectively.

**Table 1.** Demographic characteristics of the studied HCPs.

	***F* (%)**
**Age groups**	
18 to <35 years	460 (56.37)
35 to <50 years	254 (31.13)
50 to <65 years	61 (7.48)
65 years or more	41 (5.02)
**Sex**	
Female	422 (51.72)
Male	394 (48.28)
**Marital status**	
Single	390 (47.79)
Divorced/widow	73 (8.95)
Married	353 (43.26)
**Nationality**	
Cameroonian	157 (19.24)
Egyptian	183 (22.4)
Saudi	177 (21.69)
Syrian	234 (29.7)
Others^a^	65 (7.97)
**Place of residency**	
Rural	144 (17.65)
Urban	672 (82.35)
**Specialty**	
Administrative role at health care services	30 (3.68)
Dentist	34 (4.17)
Nurse	92 (11.27)
Other health care professions	102 (12.50)
Non-health care profession	193 (23.67)
Pharmacist	48 (5.88)
Physical therapist	17 (2.08)
Physician	281 (34.44)
Student	17 (2.08)
Veterinarian	2 (0.25)
*Works include interaction with patients (frontline)*	304 (37.25)
**Had comorbidities**	
No	667 (81.74)
Only organic illness	136 (16.67)
Only psychiatric (mental) illness	6 (0.74)
Yes, both	7 (0.86)
**Chickenpox vaccination status**	
No, I hesitate	79 (9.68)
No, but I intend to get the vaccine	85 (10.42)
No, I don't intend to	342 (41.91)
Yes	310 (37.99)

^a^ Others include Iraq and Libyan.

### 3.2. Knowledge and experience of the HCPs with COVID-19

While the majority of our HCP participants (62.50%) have been infected with the SARS-CoV-2 virus. In the meantime, 55.4% had taken courses or training programmes regarding COVID-19. Regarding HSB, 38.7% got their information through passive attention, while only 28.8% got it through active search. The median total knowledge score of the participants was 11, with an IQR between 9 and 13. Most of the participants (56.86%) had a moderate level of knowledge regarding COVID-19. The HCPs' knowledge and experience with COVID-19 are summarised in Table 2.

**Table 2.** The HCPs' knowledge and experience.

Participants' percentages of correct answers regarding	COVID-19 *F* (%)	Monkeypox *F* (%)
**You got infected with**	510 (62.50)	26 (3.19)
**You dealt with cases or suspected cases**	590 (62.50)	34 (4.17)
**You received a program or training course**	452 (55.39)	72 (8.82)
**Have you been vaccinated**		
No, I hesitate	20 (2.45)	187 (22.92)
No, but I intend to get the vaccine	44 (5.39)	158 (19.36)
No, I don't intend to	103 (12.62)	423 (51.84)
Yes	649 (79.53)	48 (5.88)
You ever sexually contacted homosexual people(MSM)		72 (8.82)
**Which of the following is true regarding**		
Fever	668 (81.86)	393 (48.16)
Headache	665 (81.50)	350 (42.89)
Cough	657 (80.51)	293 (35.91)
Sore throat	647 (79.29)	338 (41.42)
Skin lesion	278 (34.07)	431 (52.82)
Back pain	250 (30.64)	239 (29.29)
Muscle pain	168 (20.59)	275 (33.70)
Lymph node swelling	464 (56.86)	260 (31.86)
Has a post-COVID-19 symptoms (multi-system affection)	504 (61.76)	289 (35.42)
The incubation period is less than a week	366 (44.85)	313 (38.36)
The incubation period is between a week and two weeks	332 (40.69)	220 (26.96)
Is a zoonotic disease	345 (42.28)	353 (43.26)
Can be transmitted by droplets	650 (79.66)	218 (26.72)
Can be a sexually transmitted disease	489 (59.93)	299 (36.64)
Can be transmitted by contact with contaminated surfaces	210 (25.74)	252 (30.88)
Can lead to death	673 (82.48)	365 (44.73)
Is a viral infection	192 (23.53)	528 (64.71)
Is a bacterial infection	158 (19.36)	232 (28.43)
Has a known treatment	130 (15.93)	264 (32.35)
Has a known vaccine	658 (80.64)	532 (65.20)
Is an emerging disease	407 (49.88)	321 (39.34)
Is a re-emerging disease	128 (15.69)	528 (64.71)
**The level of knowledge**		
High	46 (5.64)	37 (4.53)
Moderate	464 (56.86)	309 (37.87)
Poor	306 (37.50)	470 (57.60)
**Total knowledge score, median (IQR)**	11.00(9.00–13.00)	9.00 (5.00–13.00)

The multivariate linear regression analysis of the COVID-19-K score revealed some significant associations with independent variables (Table 3). HCPs who were aged 65 years of age or older are predicted to have a 1.3-point higher score of knowledge compared to those with ages between 18 and less than 35 years of age (*p* value = 0.026). Moreover, being married increases the score by 0.54 points (*p* value = 0.042). Similarly, infection with COVID-19 and receipt of training programs or courses increase the knowledge score by 1.54 and 1.39, respectively (*p* value <0.0001). Adherence to health precautions slightly increases the overall COVID-19-K score (0.058 points; *p* value = 0 .001).

**Table 3.** Multivariate linear regression analysis of COVID-19 knowledge score.

Item	Estimate	Std. error	*t* value	*p* value
**(Intercept)**	2.967	0.627	4.730	<0.001*
***Age group*: (18 to <35 years)**				
35 to <50 years	−0.097	0.266	−0.363	0.716
50 to <65 years	0.252	0.452	0.557	0.578
65 years or more	1.282	0.574	2.234	0.026*
***Sex*** (female)				
Male	−0.280	0.202	−1.389	0.165
***Marital status*** (single)				
Divorced/widow	−0.057	0.435	−0.131	0.896
Married	0.536	0.263	2.033	0.042*
***Place of residence*** (rural)				
Urban	−0.200	0.264	−0.760	0.447
***Does your work include interaction with patients?** (frontline)*				
Yes	0.176	0.229	0.769	0.442
***Do you suffer from any organic or psychiatric (mental) illnesses**?(No)*				
Only organic illness	0.018	0.319	0.058	0.954
Only psychiatric (mental) illness	1.879	1.118	1.681	0.093
Yes, both	0.005	1.063	0.004	0.997
***Have you been vaccinated with COVID-19 vaccine?(No)***				
Yes	−0.103	0.263	−0.394	0.694
***Have you got infected with SARS-CoV-2 virus? (No)***				
Yes	1.537	0.238	6.462	<0.001*
***Have you received any program or training course on COVID-19? (No)***				
Yes	1.388	0.236	5.875	<0.001*
***Knowledge-seeking behaviour (information on COVID-19**) (passive attention)*				
Active search	0.224	0.263	0.850	0.396
Ongoing search	−0.490	0.340	−1.439	0.150
Passive search	−0.055	0.280	−0.196	0.845
*Animal exposure pattern score*	−0.042	0.022	−1.942	0.053
*Adherence to health precautions*	0.058	0.017	3.366	0.001*

*Significance level is *p* value <0.05.

### 3.3. Knowledge and experience of the HCPs with MPOX

Around 3.2% of the HCPs were infected with MPOX, while 4.2% have dealt with suspected cases of MPOX. In the meantime, only 8.8% had courses or training programs regarding MPOX. Noteworthy, 58 HCPs (7.1%) have connected sexually with homosexual people.

The median total MPOX-K score of the HCPs was 9, with an IQR between 5 and 13. Most of the HCPs (57.60%) had a poor level of knowledge regarding MPOX. Knowledge and experience of the HCPs with MPOX are summarized in Table 2.

Regarding the regression analysis of the MPOX-K score, people aged 65 or more were significantly associated with a decrease in the MPOX-K score by 3.6 points (*p* value <0.0001). Similarly, HCPs with only organic illnesses are indicated to have a lower score by 1.84 points (*p* value <0.0001).

On the other hand, HCPs working on the frontlines with patients and those who had training programs or courses are shown to have a higher score by 1.2 and 3.2 points, respectively (*p* value <0.0001). Noteworthy, HCPs who had a history of sexual contact with homosexual people were associated with a higher score by 2.48 points (*p* value <0.0001). In addition, each point increase in the animal contact pattern scale was associated with a decrease in knowledge score of 0.12 (*p* value <0.0001). The results of the multivariate regression analyses are detailed in Table 4.

**Table 4.** Multivariate linear regression analysis of monkeypox knowledge score.

Item	Estimate	Std. error	*t* value	*P v*alue
**(Intercept)**	5.271	0.801	6.579	<0.001*
***Age group*** (18 to <35 years)				
35 to <50 years	0.391	0.438	0.892	0.373
50 to <65 years	0.125	0.735	0.170	0.865
65 years or more	−3.574	0.932	−3.835	<0.001*
***Sex*** (female)				
Male	−0.460	0.321	−1.433	0.152
***Marital status** (single)*				
Divorced/widow	0.760	0.714	1.064	0.288
Married	1.132	0.431	2.627	0.009*
***Place of residence*** (rural)				
Urban	−0.693	0.430	−1.610	0.108
***Does your work include interaction with patients** ?(frontline)*				
Yes	1.211	0.365	3.316	0.001*
***Do you suffer from any organic or psychiatric (mental) illnesses? (No)***				
Only organic illness	−1.840	0.520	−3.536	<0.001*
Only psychiatric (mental) illness	2.093	1.838	1.138	0.255
Yes, both	−0.124	1.744	−0.071	0.943
***Have you been vaccinated with the chickenpox vaccine? (No)***				
Yes	−0.609	0.360	−1.689	0.092
***Have you been vaccinated with the monkeypox vaccine? (No)***				
Yes	−1.119	0.720	−1.555	0.120
***Have you got infected with monkeypox virus?(No)***				
Yes	0.085	1.022	0.083	0.934
***Have you dealt with cases or suspected cases of monkeypox? (No)***				
Yes	0.944	0.886	1.064	0.287
***Have you received any program or training course on monkeypox? (No)***				
Yes	3.178	0.582	5.457	<0.001*
***Have you ever sexually contacted homosexual people? (No)***				
Yes	2.489	0.654	3.806	<0.001*
***Knowledge-seeking behaviour (information on Monkeypox**) (passive attention)*				
Active search	1.265	0.454	2.787	0.005*
Ongoing search	1.576	0.616	2.557	0.011*
Passive search	0.411	0.403	1.019	0.308
*Animal exposure pattern score*	−0.119	0.034	−3.530	<0.001*
*Adherence to health precautions*	0.029	0.028	1.019	0.308

*Significance level is *p* value <0.05.

### 3.4. The source of information and the knowledge-seeking behaviour

**Figure 1.**
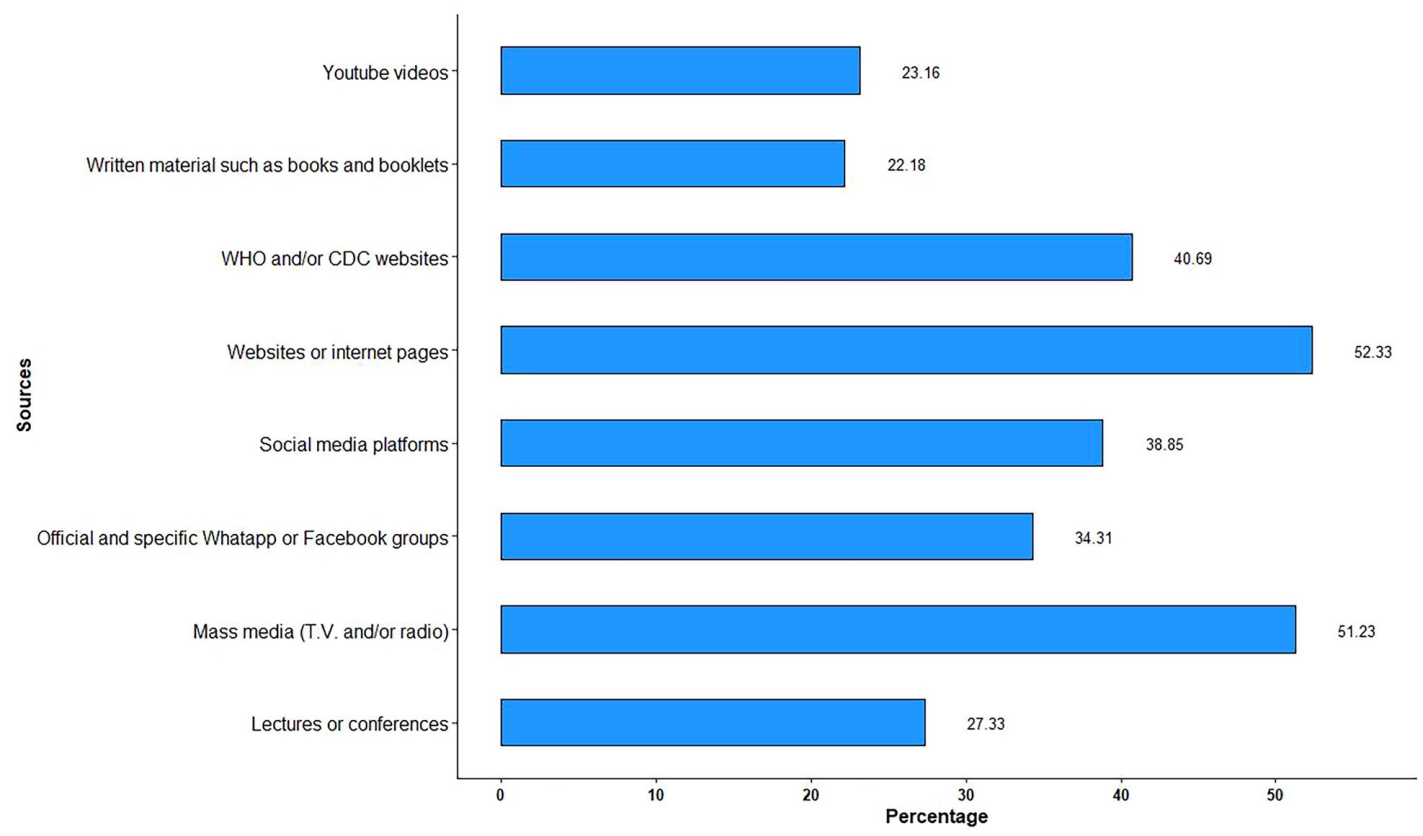
**Sources of information about monkeypox**.

Websites, followed by mass media, were the most commonly used sources of information. Sources of information for MPOX are plotted in Figure 1.

**Figure 2.**
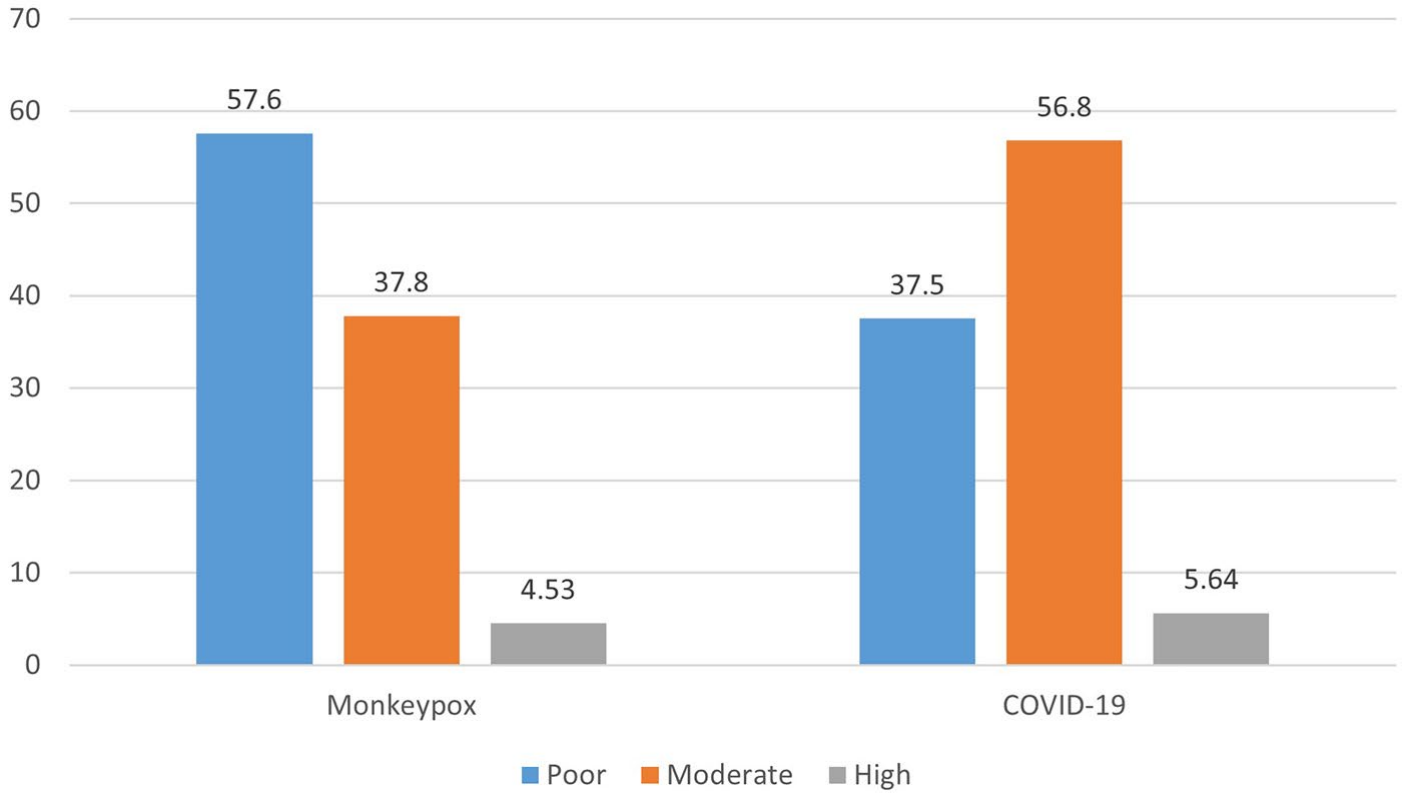
**The levels of knowledge among HCPs regarding COVID-19 and MPOX**.

Regarding MPOX-KSB, 50.9% were achieved through passive attention, while only 18.01% and 23.04% got their information through active and passive search, respectively. Knowledge-seeking behaviour was significantly associated with knowledge scores for both COVID-19 and MPOX (Table 5). Comparisons between knowledge levels of COVID-19 and MPOX are shown in Figure 2.

**Table 5.** The HCPs' knowledge-seeking behaviour regarding COVID-19 and monkeypox.

Knowledge-seeking behaviour	COVID-19		Monkeypox	
	*F* (%)	Median (IQR)	*F* (%)	Median (IQR)
Passive attention	316 (38.73)	11.00(9.00–13.00)	415 (50.86)	8.00(3.00–13.00)
Active search	235 (28.80)	11.00(10.00–15.00)	147 (18.01)	10.00(8.00–14.00)
Ongoing search	103 (12.62)	12.00(10.00–14.00)	66 (8.09)	12.00(8.00–15.00)
Passive search	162 (19.85)	11.00(9.00–13.00)	188 (23.04)	9.00(7.00–13.00)
*P v*alue of Kruskal–Wallis test	0.001*		<0.001*	

^*^ Significant *p* value <0.05.

**Table 6.** The HCPs' belief about MPOX and their adherence to health precautions (MPOX health belief model).

	**Strongly agree** ***F* (%)**	**Agree** ***F* (%)**	**Neutral** ***F* (%)**	**Disagree** ***F* (%)**	**Strongly disagree** ***F* (%)**
***(A) Perceived susceptibility and severity***					
Do you consider yourself liable to infection with monkeypox? (perceived susceptibility) Mean (SD) 2.91 (1.00)	40 (4.90)	185 (22.55)	330 (40.44)	190 (23.28)	72 (8.82)
Do you consider yourself liable to severe infection with monkeypox? (perceived severity) Mean (SD) 2.56 (0.99)	29 (3.55)	85 (10.42)	324 (39.71)	2 53 (31.00)	125 (15.32)
***(B) Cues to action (what is your expectation regarding the spread of monkeypox in comparison to COVID-19?***					
As COVID-19 Less than COVID-19 More than COVID-19	81 (9.93) 715 (87.62) 20 (2.45)				
***(C) Action (adherence to health precautions)***					
	**Always** ***F* (%)**	**Often** ***F* (%)**	**Sometimes** *F* (%)	**No or rarely** *F* **(%)**	**Total practice**
Wearing masks	106 (12.99)	171 (20.96)	340 (41.67)	199 (24.39)	Median= 18.00 (IQR) = (15.00–23.00)
Keeping safe spaces or distances	99 (12.13)	139 (17.03)	334 (40.93)	244 (29.90)	
Regular disinfection of surfaces	113 (13.85)	163 (19.98	360 (44.12)	180 (22.06)	
Hygienic hand wash	300 (36.76)	263 (32.23)	162 (19.85)	91 (11.15)	
Sticking to cough etiquette	243 (29.78)	177 (21.69)	218 (26.72)	178 (21.81)	
Drinking more than two liter of water daily	204 (25.00)	203 (24.88)	279 (34.19)	130 (15.93)	
Supplements intake	85 (10.42)	137 (16.79)	293 (35.91)	301 (36.89)	
Regular exercise one hour and a half per week	90 (11.03)	152 (18.63)	312 (38.24)	262 (32.11)	

**Always** (seven days per week), **often** (5–6 days per week), **sometimes** (2–4 days per week) and **no or rarely** (less than once per week).

### 3.5. The HCPs' beliefs about MPOX and their adherence to preventive and promotive health precautions (are shown in Table 6)

The majority of the participants (87.6%) thought that the MPOX will be less than that of COVID-19. Regarding health precautions in the past two weeks, hand hygiene had the highest percentage of HCPs that were always doing so (*n* = 300, 36.8%) followed by sticking to cough etiquette (*n* = 243, 29.8%). On the other hand, supplemental intake had the highest percentage of HCPs that never or rarely did so (*n* = 301, 36.9%) followed by regular exercise of one and a half hours per week (*n* = 262, 32.1%).

### 3.6. HCPs' animal contact patterns

The total score of the HCPs' animal contact patterns had a median (IQR) of 2.00 (0.00–6.00). Rodents, mice and hamsters had the highest daily contact patterns (9.93%), followed by aquatic pets such as fish (8.33%). As for weekly contact patterns, avian pets such as parrots and passerines were the most (36.76%). Monkeys, followed by camels and bats, had the least contact patterns, as 84.44%, 82.97% and 79.29% of our HCPs had not been exposed to these animals at all, respectively. The HCPs' animal contact patterns are detailed in Table 7.

**Table 7.** Animal contact patterns among the HCPs.

	Not at all *F* (%)	Yearly or monthly *F* (%)	Weekly *F* (%)	Daily *F* (%)
Rodents, mice,and hamsters	550 (67.40)	41 (5.02)	144 (17.65)	81 (9.93)
Bats	647 (79.29)	32 (3.92)	71 (8.70)	66 (8.09)
Monkeys	689 (84.44)	17 (2.08)	57 (6.99)	53 (6.50)
Camels	677 (82.97)	16 (1.96)	63 (7.72)	60 (7.35)
Reptile pets such as turtles, alligators, crocodiles, lizards and snakes	645 (79.04)	27 (3.31)	94 (11.52)	50 (6.13)
Aquatic pets such as fish	603 (73.90)	27 (3.31)	118 (14.46)	68 (8.33)
Avian pets such as parrots, and passerines	390 (47.79)	73 (8.95)	300 (36.76)	53 (6.50)
**Total animal contact patterns score**	**Median (IQR)** = 2 (0–6)			

**Table 8.** Spearman's correlation analysis

Variable 1 (total score of)	Variable 2 (total score of)	*r*	*p* Value
Monkeypox knowledge	COVID-19 knowledge	0.48	<0.001*
Monkeypox knowledge	Animal contact patterns	−0.20	<0.001*
COVID-19 knowledge	Animal contact patterns	−0.33	<0.001*
Monkeypox knowledge	Adherence to health precautions	0.1	0.005*
COVID-19 knowledge	Adherence to health precautions	0.16	<0.001*
Animal contact patterns	Adherence to health precautions	−0.03	0.374

^*^ The significance level is *p* value <0.05/*r* (correlation coefficient).

### 3.7. Spearman's correlation analysis (are shown in Table 8)

Spearman's correlation analysis revealed a highly significant correlation between the total scores of COVID-19-K and MPOX-K score (r = 0.48, *p* value <0.0001). On the other hand, the COVID-19-K and animal pattern of exposure also had a significantly negative correlation (r = −0.33, *p* value <0.0001). There was also a negative correlation between MPOX-K score and the animal contact patterns (r = −0.2, *p* value <0.0001).

## 4. Discussion

Epidemiologic research has definitely shown that HCPs' interactions with other groups play significant roles in the prevention of various infectious diseases and affect their behaviour [25]. We reported in September 2022 that HCPs from the analysed countries knew less about the MPOX disease than they did about COVID-19. The total COVID-19-K and MPOX-K scores were highly significant, correlated with one another, and significantly related to KSB. The MPOX-K score and the animal contact patterns score had a negative connection.

### 4.1. COVID-19-knowledge and experience among HCPs

The majority of HCPs (56.66%) had a moderate level of knowledge about COVID-19, which can be attributed to the fact that only 55.39% had courses or training programs in this area. In addition, regarding their KSB, 38.73% was gained through passive attention, while only 28.8% did so through active search.

The level of knowledge varies depending on the target population, the time and the location; e.g., among HCP respondents in Aseer (December 2020), 412 (84%) had good knowledge, while 79 (16%) exhibited inadequate knowledge [26], whereas the level of adequate knowledge in Cameroon, June 2020, was 84.2% [27], among the public in Egypt was 80.9%, and in India it was 83% [21]. However, this finding is higher than that among 33.9% of chronic patients in Ethiopia [28].

The multivariate linear regression analysis of the COVID-19-K score in our study found several significant correlations with independent factors (e.g., age, marital status, and training), as it was significantly higher among HCPs (age 65 year and older) compared to those ages between 18 and less than 35 y (OR = 1.3, *p* value = 0.025). As well as being married (OR = 0.55, *p* value = 0.039) and receiving training or attending courses or training programmers (OR = 1.54 and 1.38, respectively, *p* value = 0.0001). In agreement with Ngwewondo et al., who reported that for public health policymakers and HCPs to identify the target audience for COVID-19 prevention and sensitization, it will be helpful to consider the factors of gender, age and city of residency that positively connect with knowledge and practice of the disease [27].

Regarding the lowest COVID-19-K score, women HCPs were 88% more likely than men to have insufficient knowledge (OR 1.88; 95% CI: 1.44–2.44). Additionally, it was lowest among HCPs with 1–3 years of experience (OR 2.05; 95% CI: 1.49–2.82). All parameters that were significant in univariate logistic regression were included in multivariate logistic regression, and their relationship to knowledge levels was evaluated. The relationship between the variables of gender (aOR 1.55: 95% CI: 1.15–2.09), educational level (aOR 2.51: 95% CI: 1.64–3.83), and employment history (aOR 1.47: 95% CI: 1.01–2.15) persisted as being significant. In line with Alduraywish et al., who stated that univariate logistic regression revealed that age, gender, country, and location were significantly linked with knowledge, there were significant relationships between the level of education and the occupation-related elements, e.g., the workplace, the frontline or not, and years of experience [28].

### 4.2. MPOX-K and experience among HCPs

In our study, most (57.6%) HCPs reported a poor level of knowledge; this can be attributed to the fact that the majority of the studied HCPs were from non-MPOX-endemic countries (Syria, Egypt and Saudi Arabia), and only 8.8% had courses or training programmes regarding MPOX. Moreover, 50.9% of their KSBs were through passive attention, while only 18.01% and 23.04% were through active and passive search, respectively. Websites, followed by mass media, were the most commonly used sources of information. In addition, only a small percentage was risky; for instance, around 3.2% were infected with MPOX, while only 4.2% have dealt with suspected cases of MPOX. In the meantime, it is noteworthy that 58 HCPs (7.1%) have connected sexually with homosexual people.

Nearlyall the published studies showed that most participants had an insufficient level of MPOX knowledge, but this varies depending on the target population, the time, the nature of the questions, and the location. In this multicounty cross-sectional study, most of the HCPs (57.6%) had a poor level of knowledge. Nearly, similar results were reported in another international study among HCPs (55%) who had insufficient information about it [22]; in Saudi Arabia, among physicians, it was 57.6% [29]; and while among the general public in Indonesia, it was 63.5% [30], among the general public in Malaysia, it was 40.7%) (40.7%) [31].

From investigations of knowledge-related variables using single- and multiple-variable logistic regression, these results demonstrate a relationship between increased understanding of MPOX and higher levels of education, particularly among HCPs.

According to our regression analysis results of the MPOX-K score, in contrast to the COVID-19-K score, HCPs aged 65 year or older were significantly associated with a decrease in the MPOX-K score (OR = 3.6, *p* value <0.0001), HCPs with only organic illnesses (OR = 1.87, *p* value .0001), while the frontline HCPs (OR = 1.25, *p* value <0.0001), HCPs who had training programs or courses (OR = 3.18, *p* value <0.0001), and HCPs who had a history of sexual contact with homosexual people (O*R* = 2.4, *p* value <0.0001), being a female doctor and working in the private sector (OR: 2.08; 95% CI: 1.38–3.12 and OR: 2.78; 1.52–5.10), respectively, are shown to have a higher MPOX-K score. Similar to what Alshahrani et al. found, a higher level of MPOX-K was strongly linked to a number of demographic factors, including age, gender, marital status and conditions related to work, in the first logistic regression analysis [29].

### 4.3. The HCPs' belief about MPOX and their adherence to health precautions

In this study, 715 (87.62%) of HCPs reported that MPOX was less harmful than COVID-19, while in another study in the MENA region in August 2022, 48.4% of HCPs believed that MPOX could cause an epidemic and a tremendous burden comparable to that of COVID-19 [19], while in a Saudi Arabian study, only 25.3% of the study population was very concerned about a future MPOX pandemic, while 48.7% had no to little concern. Additionally, compared to COVID-19 [25], MPOX disturbed 18.1% of participants more. In a survey of the general public among five Arabic countries in the Middle East in August 2022, COVID-19 generated more anxiety (62%) than MPOX [32].

Most HCPs reported that 423 (51.84%) did not vaccinate and did not intend to be vaccinated. Moreover, the median of total adherence to the preventive and promotional health behaviour score was 2 (low), with a range of 0–6. The majority of them had a low level of knowledge, and they also believed that MPOX was a severe disease and that they were not susceptible to it, which can explain this. Vaccine hesitancy is a widespread challenge, fuelled by misinformation and mistrust, particularly in rural areas that have many determinants, including several aspects such as structural (e.g. government, country), extrinsic (e.g., family, friends), intrinsic (e.g., self-perception), and other factors (financial and nonfinancial) [33, 34].

### 4.4. The animal contact pattern among HCPs

Regarding the animal contact pattern among the studied HCPs, in descending order either on a daily or weekly basis , they were: avian pets such as parrots and passerines (353; 43.2%); rodents, mice, and hamsters (225; 27.6%); aquatic pets such as fish (186; 22.8%); reptile pets such as turtles, alligators, crocodiles, lizards and, snakes (144; 17.6%); and bats (137; 16.8%). This may be attributed to pets'essential role in human health and life, including the HCPs, through improvements in all dimensions of health (social, physical, spiritual, mental and psychological wellbeing) [35]. In addition to other forms of contact with or handling of animals during patient care, in certain health care settings, animals may be involved in therapy or assistance programmers, such as animal-assisted therapy or service animals. Laboratory work: HCPs are involved in laboratory work [36].

Scientists have long warned of the dangers of 'zoonotic spillovers,' or the human-animal transmission of viruses, and estimate that 70% of recently emerging infectious diseases have been transmitted from animals to people. The relatively high animal contact pattern among HCPs can help explain this [37].

Therefore, worldwide, HCPs face many new challenges, such as the diagnosis, therapy, and prevention of any new emerging or remerging diseases, e.g., MPOX, particularly in nations where cases have not yet been documented [30]. To contain the rapid spread of MPOX outbreaks in countries since May 14, 2022, an effective strategy for deeper comprehension, preventing, and managing future pandemics could involve the establishment of a comprehensive Global One-Health initiative. Moreover, WHO has delineated six crucial domains for pandemic preparedness: risk assessment, surveillance, laboratory capability, clinical care, public health actions ,and risk communication [18, 38].

### 4.5. Limitations and Strengths

The response rate varies across different countries to be highest in Syria, which had the highest response percentage of 29.7%, followed by Egypt, and Saudi Arabia with percentages of 22.6% and 21.69%, respectively, while Cameroon had the lowest response percentage of 19.2%. This can be attributed to the language barrier as only two regions out of 10 in Cameroon—about 17% of Cameroon's population—are mainly anglophones. We checked their validity to make sure the questions they employed were true to the subject of the study.

Cross-sectional research may be carried out quickly and inexpensively, but it must take into account the particular and reliable causal relationship as well as the prevalence of MPOX. Regarding the online cross-sectional study, it is particularly challenging to get replies from those who do not have the time to complete the survey, do not have a mobile phone and a poor Internet connection, or are unable to do so due to technical issues.

This study had many strengths aside from its relative large size, as it included 816 HCPs from four countries: three non-MPOX-endemic countries (Syria, Egypt and Saudi Arabia) and one MPOX-endemic country (Cameroon). Moreover, this study explored the animal contact patterns, KSB and MPOX health belief models (HBMs) among HCPs.

## 5. Conclusions

Most HCPs reported that passive attention was their KSB regarding MPOX and COVID-19. Most of them showed that their level of knowledge was low regarding MPOX and moderate regarding COVID-19. The regression analysis of the MPOX-K score revealed that HCPs who were female doctors working in the private sector, had chronic diseases, worked on the frontlines with patients, and had training programmes or courses had a higher score.

Regarding the animal contact patterns among the studied HCPs, in descending order either on a daily or weekly basis were: avian pets such as parrots and passerines; rodents, mice and hamsters; aquatic pets such as fish; reptile pets such as turtles, alligators, crocodiles, lizards and snakes; and bats.

There was a highly significant positive correlation between the total scores of COVID-19-K and MPOX-K. On the other hand, the total scores of COVID-19-K and MPOX-K scores were significantly negatively correlated with the total scores of animal contact patterns.

## Recommendation

- Finally, health authorities throughout the world have increased their efforts to ensure the control of the spread of any potential new pandemic by researching the methods of transmission and early clinical signs. Awareness, concern, and perception of the available vaccinations are significant issues during the present rise in the number of reported MPOX infections, especially among health care practitioners and medical staff members.
- Efforts to increase the perception of the danger that MPOX poses to public health may increase adherence to preventive measures.
- It is important for HCPs to take appropriate precautions to minimize the risk of animal contact, such as wearing PPE, practicing proper hand hygiene, and following established infection control protocols.
- As a result, we recommend that health care professionals be educated on the hazards of MPOX infection and the importance of vaccination through the implementation of adequate awareness and training programs.

## Data Availability

The datasets used and/or analysed during the current study are available from the corresponding author upon reasonable request (dr_samar11@yahoo.com).
